# Role of mineralization inhibitors in the regulation of hard tissue biomineralization: relevance to initial enamel formation and maturation

**DOI:** 10.3389/fphys.2014.00339

**Published:** 2014-09-10

**Authors:** Henry C. Margolis, Seo-Young Kwak, Hajime Yamazaki

**Affiliations:** ^1^Department of Applied Oral Sciences, Center for Biomineralization, The Forsyth InstituteCambridge, MA, USA; ^2^Department of Developmental Biology, Harvard School of Dental MedicineBoston, MA, USA

**Keywords:** amorphous calcium phosphate, biomineralization, dental enamel, inhibitors, mineral phase transformation

## Abstract

Vertebrate mineralized tissues, i.e., enamel, dentin, cementum, and bone, have unique hierarchical structures and chemical compositions. Although these tissues are similarly comprised of a crystalline calcium apatite mineral phase and a protein component, they differ with respect to crystal size and shape, level and distribution of trace mineral ions, the nature of the proteins present, and their relative proportions of mineral and protein components. Despite apparent differences, mineralized tissues are similarly derived by highly concerted extracellular processes involving matrix proteins, proteases, and mineral ion fluxes that collectively regulate the nucleation, growth and organization of forming mineral crystals. *Nature*, however, provides multiple ways to control the onset, rate, location, and organization of mineral deposits in developing mineralized tissues. Although our knowledge is quite limited in some of these areas, recent evidence suggests that hard tissue formation is, in part, controlled through the regulation of specific molecules that inhibit the mineralization process. This paper addresses the role of mineralization inhibitors in the regulation of biological mineralization with emphasis on the relevance of current findings to the process of amelogenesis. Mineralization inhibitors can also serve to maintain driving forces for calcium phosphate precipitation and prevent unwanted mineralization. Recent evidence shows that native phosphorylated amelogenins have the capacity to prevent mineralization through the stabilization of an amorphous calcium phosphate precursor phase, as observed *in vitro* and in developing teeth. Based on present findings, the authors propose that the transformation of initially formed amorphous mineral deposits to enamel crystals is an active process associated with the enzymatic processing of amelogenins. Such processing may serve to control both initial enamel crystal formation and subsequent maturation.

## Introduction

*Biomineralization* is the process by a wide variety of living organisms, including mollusks, sponges and unicellular diatoms, for example, produce functional mineralized tissues (Mann, 2001). Vertebrate mineralized tissues, like dental enamel, dentin, cementum, and bone, fulfill specialized functions that reflect differences in their hierarchical organization and composition (Weiner, 1986). Although each of these tissues is comprised of a crystalline calcium apatite mineral phase and a protein component, they differ with respect to overall structure, crystal size and shape, level and distribution of trace mineral ions, the nature of the proteins present, and the relative proportions of mineral and protein components. Differences in structural organization and composition give rise to mineralized tissues with different physical and mechanical properties that are well-suited for their intended biological purpose (Birchall, 1989; Currey, 1999).

Although the mechanisms by which these mineralized tissues form are not fully understood, it is apparent that the unique structure of each mineralized tissue, including dental enamel, is the result of highly concerted cell and extracellular processes that regulate the on-set, growth rate, shape, location and arrangement of forming mineral crystals (Weiner, 1986). Evidence also suggests that critical aspects of hard tissue formation are controlled, in part, through the regulation of specific molecules that inhibit mineralization. This paper addresses the role of mineralization inhibitors in the regulation of biological mineralization and the potential relevance of such mechanisms in the process of dental enamel formation (amelogenesis).

## Fundamental aspects of vertebrate mineralized tissue formation

### Extracellular protein matrix and mineralized tissue composition

Biominerals are formed utilizing similar fundamental strategies, although there are unique differences that distinguish one tissue from another, especially dental enamel. Enamel, dentin and bone are each derived from specialized cells; ameloblasts, odontoblasts and osteoblasts, respectively. These cells secrete an extracellular protein matrix that is predominantly comprised of a hydrophobic protein and smaller amounts of acidic hydrophilic molecules. In bone and dentin, the matrix is mostly collagen, while the major enamel matrix component (>90%) is amelogenin. It has been proposed that biomineralization is generally regulated through interactions between hydrophobic components, which provide a skeletal or space-filling structure (e.g., collagen in bone and dentin), and hydrophilic (acidic) molecules (e.g., phosphophoryn in dentin Veis et al., 1991; He et al., 2005) that regulate crystal nucleation and growth (Weiner, 1986; Addadi and Weiner, 1992). Considerable evidence shows that a highly-ordered pre-assembled collagen matrix serves as a template to guide subsequent mineralization, as we have previously discussed (Margolis et al., 2006). The initial collagenous matrix is mineral free and undergoes a series changes in structure and composition prior to mineralization (Weinstock and Leblond, 1973; Septier et al., 1998; Beniash et al., 2000), resulting in the formation of tissues that are 40–50% mineral and ~35% organic by volume (Nikiforuk, 1985). The protein matrix of forming enamel is similarly comprised of a predominant hydrophobic protein (amelogenin) and two key minor protein components enamelin (hydrophilic and acidic) and ameloblastin (amphiphilic and acidic). The observations that the amelogenin-null mouse (Gibson et al., 2001) exhibits a marked enamel phenotype and that enamel does not form in the absence of enamelin (Hu et al., 2008; Smith et al., 2009) or ameloblastin (Fukumoto et al., 2004; Smith et al., 2009; Wazen et al., 2009) are in agreement with the proposed general requirement for hydrophobic-hydrophilic molecular interactions in biomineral formation.

**Figure 1:**
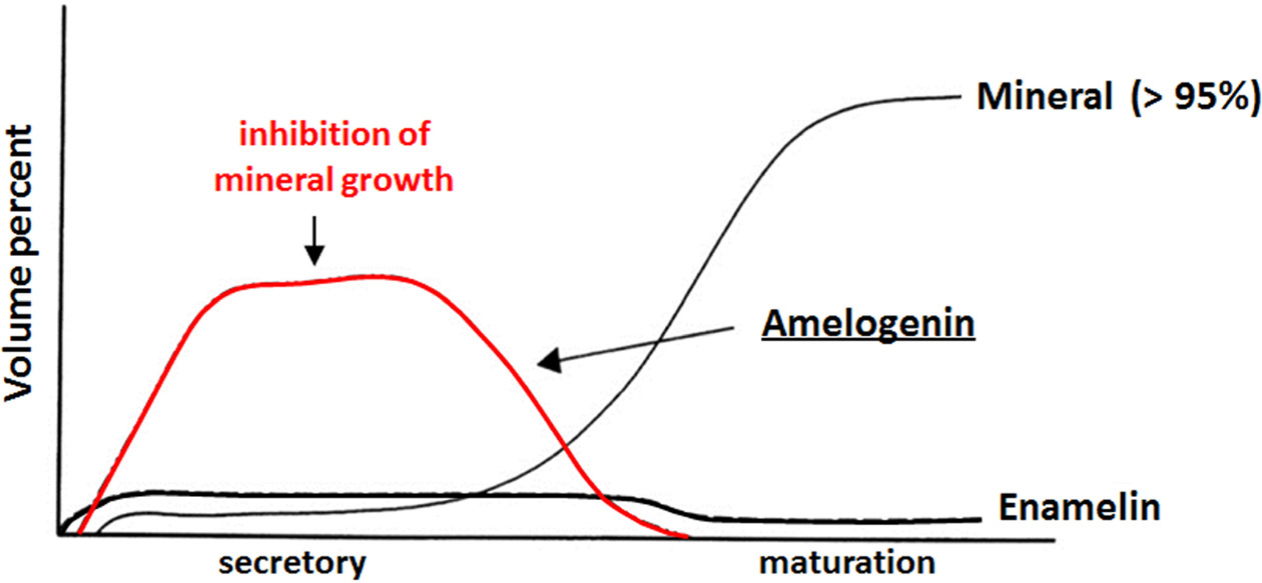
**Inverse relationship between protein and mineral content as a function of enamel development**. Amelogenin is the major component (>90%) of the extracellular enamel matrix in the secretory stage. Adapted from a figure previously published by Mann (2001), with permission from Oxford University Press. This OUP material is for view only and cannot be reused.

Despite similarities in the hydrophobic/hydrophilic composition of developing extracellular bone, dentine and enamel matrices that lead to the formation of a similar mineral phase (i.e., a carbonated hydroxyapatite), mature enamel and the mechanism of its formation differ from those of dentine and bone. First, long thin ribbons of enamel mineral begin to form almost immediately after ameloblasts lay down the enamel matrix (Nylen et al., 1963; Arsenault and Robinson, 1989; Smith, 1998), indicating that mineralization does not take place within a pre-assembled enamel matrix template, as in the case of collagen-based tissues. These long thin mineral ribbons extend hundred of microns to the full thickness of the enamel layer that is laid down during the secretory stage of amelogenesis, although the mineral component occupies only 10–20% of the enamel volume, with the remaining volume occupied by the enamel matrix and water (Robinson et al., 1988; Fukae, 2002). During the maturation stage of amelogenesis (Robinson and Kirkham, 1985), the extracellular enamel matrix is almost completely removed by resident proteases and the initially formed mineral ribbons grow in width and thickness to form a dense mineralized tissue that is >95% mineral by weight, with only 1–2 weight % of remaining protein and a small amount of water. Hence, there is an inverse relationship between the protein and mineral content during enamel development (Robinson et al., 1979), as illustrated in Figure 1. Unlike that seen in bone and dentin, the enamel matrix is transient and the resulting enamel tissue is almost fully mineralized. The transient nature of the enamel matrix is uncommon in biomineralization (Weiner, 1986).

### Calcium phosphate formation *in vitro* and *in vivo*

The formation of a calcium phosphate mineral phase *in vitro* and *in vivo* requires a condition of supersaturation with respect to the forming phase. As shown in Equation 1 for hydroxyapatite (HA: Ca_5_OH(PO_4_)_3_), a prototype for enamel, dentin and bone mineral, the degree of supersaturation (DS) can be defined as the ratio of the mean ionic activity product for a given mineral phase in solution to its thermodynamic solubility product constant (e.g., K_SP−HA_) defined in the same manner, where “()” represent the activity of the enclosed ion [e.g., (Ca^2+^_*f*_)]. The activity of an ion in solution is equal to the product of the concentration of the ion ([x^Z^_i_]) and an ion activity coefficient (a_i_) that is a function of the ion size, charge, ionic strength and temperature. Such quantities can be calculated using ion-speciation software, as we have previously described (Moreno and Margolis, 1988). Supersaturation with respect to other calcium phosphate phases, such as octacalcium phosphate [OCP: Ca_4_H(PO_4_)_3_] and dicalcium phosphate dihydrate (DCPD: CaHPO_4_^.^2H_2_O), can be defined similarly as indicated in Equations (2) and (3), respectively. A minimum requirement for mineral formation *in vitro* or *in vivo* is the condition of supersaturation. As indicated by Equations 1–3, DS is a function of calcium, phosphate and hydrogen ion activities.

(1) DSHA=[(Caf2+)5(OH−)(PO43−)3]1/9KSP−HA

(2) DSOCP=[(Caf2+)4(H+)(PO43−)3]1/8KSP−OCP

(3) DSDCPD=[(Caf2+)(HPO42−)]1/2KSP−DCPD

where, (X^*z*^_*i*_) = a_*i*_ × [X^*z*^_*i*_] and Ca^2+^_*f*_ is *free* calcium.

**Figure 2:**
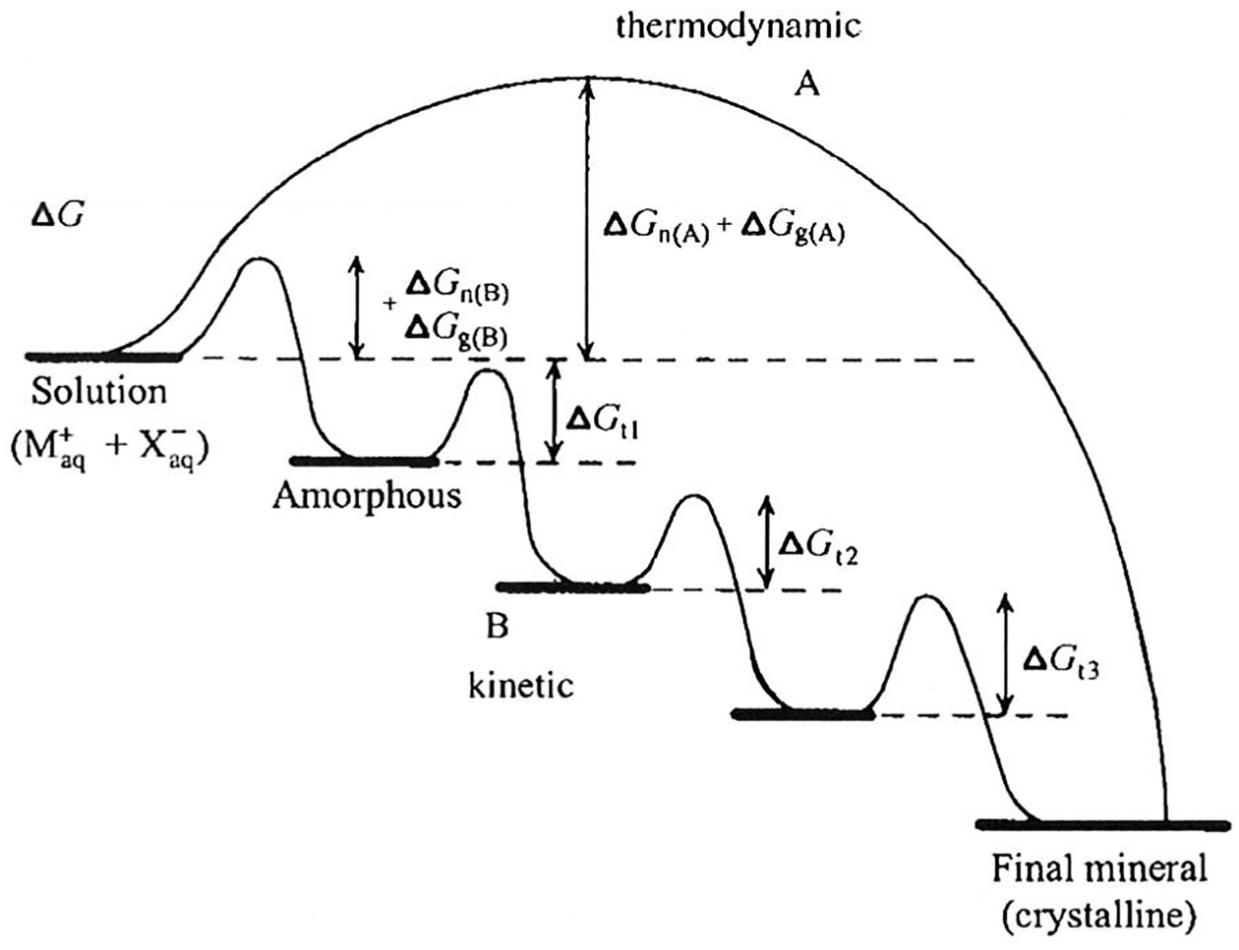
**Crystallization pathways under thermodynamic and kinetic control, illustrating that mineral formation may go through multiple phases (path B) before reaching the stable final crystalline product (e.g., HA), as discussed in the text, depending on the free energy of activation (ΔG) associated with nucleation (n), growth (g), and phase transformation (t)**. As shown, amorphous phases are frequently observed first under kinetically controlled conditions. This figure is from a publication (Colfen and Mann, 2003) in which this phenomenon is discussed in greater detail and is reproduced with permission. Copyright Wiley-VCH Verlag GmbH & Co. KGaA.

Regarding the mineralization process, a given solution can be supersaturated with respect to more than one calcium phosphate phase at the same time. Due, in part, to kinetic factors (for review, see Mann, 2001), however, the more soluble phase will form first and, through a series of *phase transformations*, convert to HA, which is the most thermodynamically stable calcium phosphate phase of the group. This phenomenon, called the *Ostwald-Lussac law of stages*, occurs in nature, as it does *in vitro* (Eanes, 2001). For example, it has been shown (Schroeder, 1969) that DCPD and OCP are the first mineral phases seen in forming dental calculus that subsequently convert to HA over time. Consistent with this observation and *Ostwald-Lussac law of stages*, at physiological pH (pH 7) and ionic strength (163 mM), HA has a relatively low solubility (expressed as the concentration of dissolved Ca^2+^) of 0.11 mM, in comparison to OCP (1.42 mM) and DCPD (2.09 mM), as calculated (Moreno and Margolis, 1988). Such transformations may go through multiple phases before reaching the stable HA phase, depending on the free energy of activation (ΔG) associated with nucleation (n), growth (g), and phase transformation (t), as previously discussed and illustrated in Figure 2 (Colfen and Mann, 2003).

**Figure 3:**
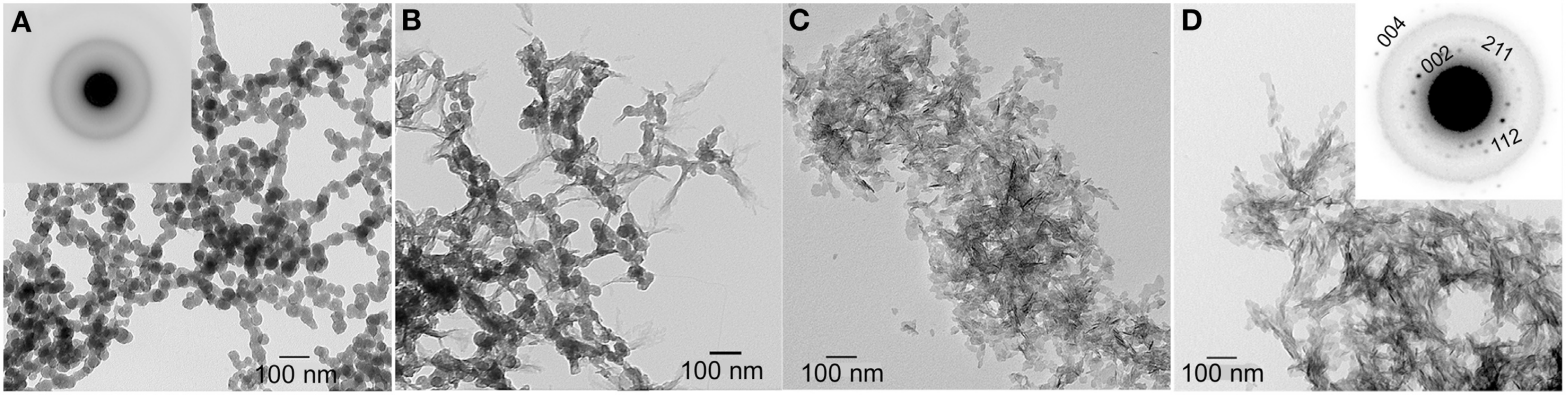
**TEM micrographs of calcium phosphate mineral products formed in the absence (control) of protein examined at selected times: 15 min (A), 45 min (B), 1–4 h (C), and 1 d (D)**. As shown at 15 min **(A)**, amorphous calcium phosphate (ACP) was initially formed based on the observed (inset) selected area electron diffraction (SAED) pattern. At 45 min, ACP phase transformation could be seen. After 1–4 h, randomly arranged plate-like apatitic crystals were found and confirmed by SAED analyses (**D**, inset). This image was reproduced from Kwak et al. (2009).

Based on the discussion above, the role of *precursor* phases involved in HA and biomineral formation has received considerable attention. The calcium phosphate mineral phases discussed so far are crystalline in nature, as confirmed using spectroscopic (FT-IR, Raman) and diffraction (X-ray and electron diffraction) methods. However, it has been known for many years (for review, see Eanes, 2001) that amorphous calcium phosphate (ACP) is the first mineral phase to form upon mixing sufficiently high concentrations of calcium and phosphate. ACP is more soluble than the noted crystalline calcium phosphates and is the kinetically favored product (Meyer and Eanes, 1978), although it readily undergoes phase transformation to HA, as shown in Figure 3. The presence of ACP in vertebrate mineralized tissues (Posner and Betts, 1975) has been a topic of considerable debate (Termine and Posner, 1966; Boskey, 1997; Eanes, 2001; Weiner, 2006) because of its transient nature. However, recent evidence suggests that the transient nature of an amorphous mineral phase, that is, the formation of an amorphous mineral phase and its subsequent transformation to a crystalline solid, is a key element in biological mineral formation (Beniash et al., 1997; Weiner et al., 2005; Deshpande and Beniash, 2008; Kwak et al., 2009; Margolis and Beniash, 2010; Wiedemann-Bidlack et al., 2011). More recently, the importance of sequential steps involving ACP formation and transformation has been confirmed *in vitro* (Habraken et al., 2013), showing that the existence of pre-nucleation clusters that precede ACP formation lowers the energy barrier to nucleation and makes amorphous phases “accessible” under conditions that would favor the formation of more stable crystalline phases. Hence, it appears calcium phosphate formation *in vivo* and *in vitro* similarly proceed through a sequence of phase transformations, potentially involving: ACP → DCPD → OCP → HA. As will be discussed below, however, matrix molecules can dramatically influence the phase transformation process.

## Role of mineralization inhibitors in biological mineralization

### Stabilization of supersaturation *in vivo*

**Figure 4:**
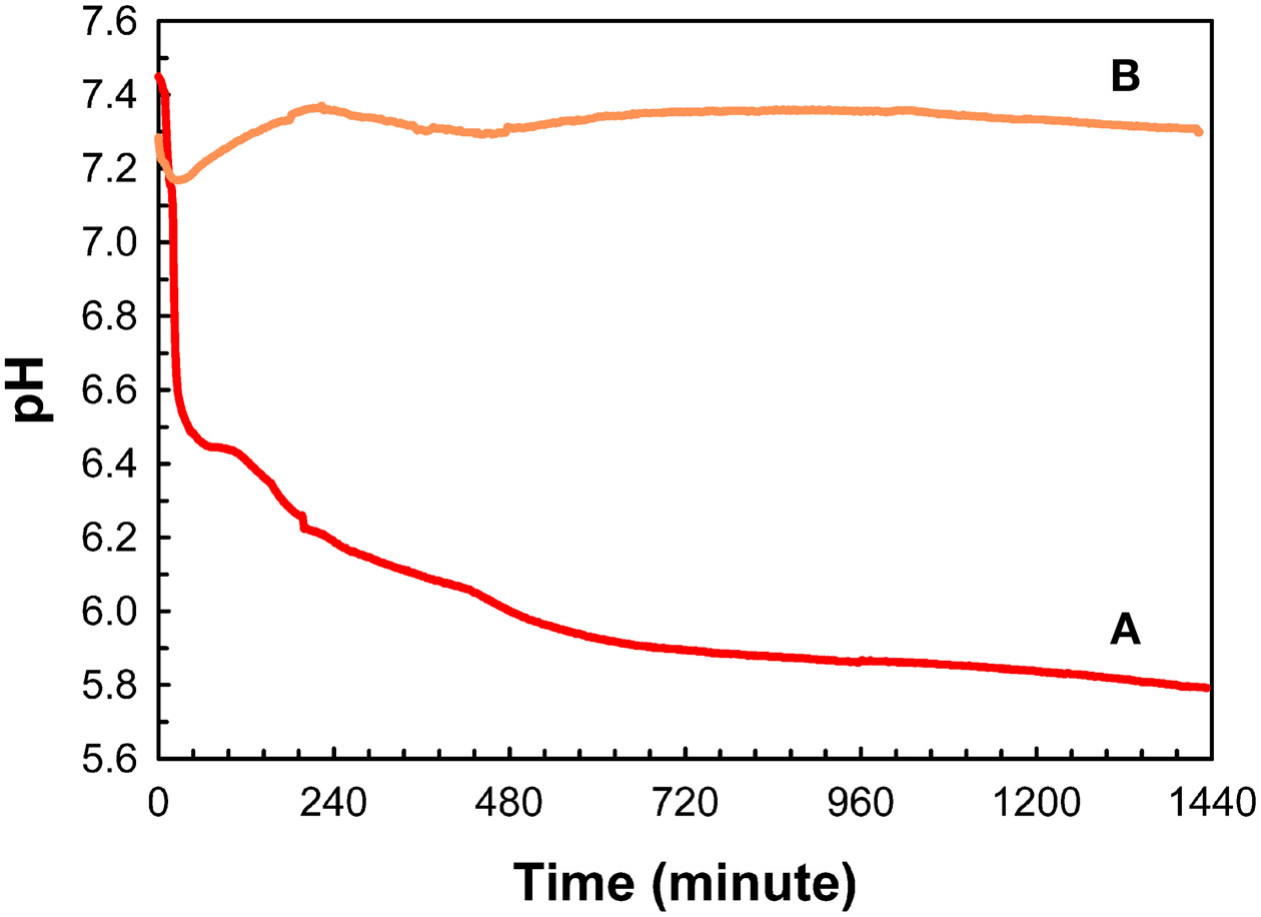
**Changes in pH as a function of time observed during mineralization experiments carried out in 60 μL sample volumes, in the absence (A) and presence of native full-length amelogenin, P173, at 2 mg/mL (B)**. This image was reproduced from Wiedemann-Bidlack et al. (2011).

**Figure 5:**
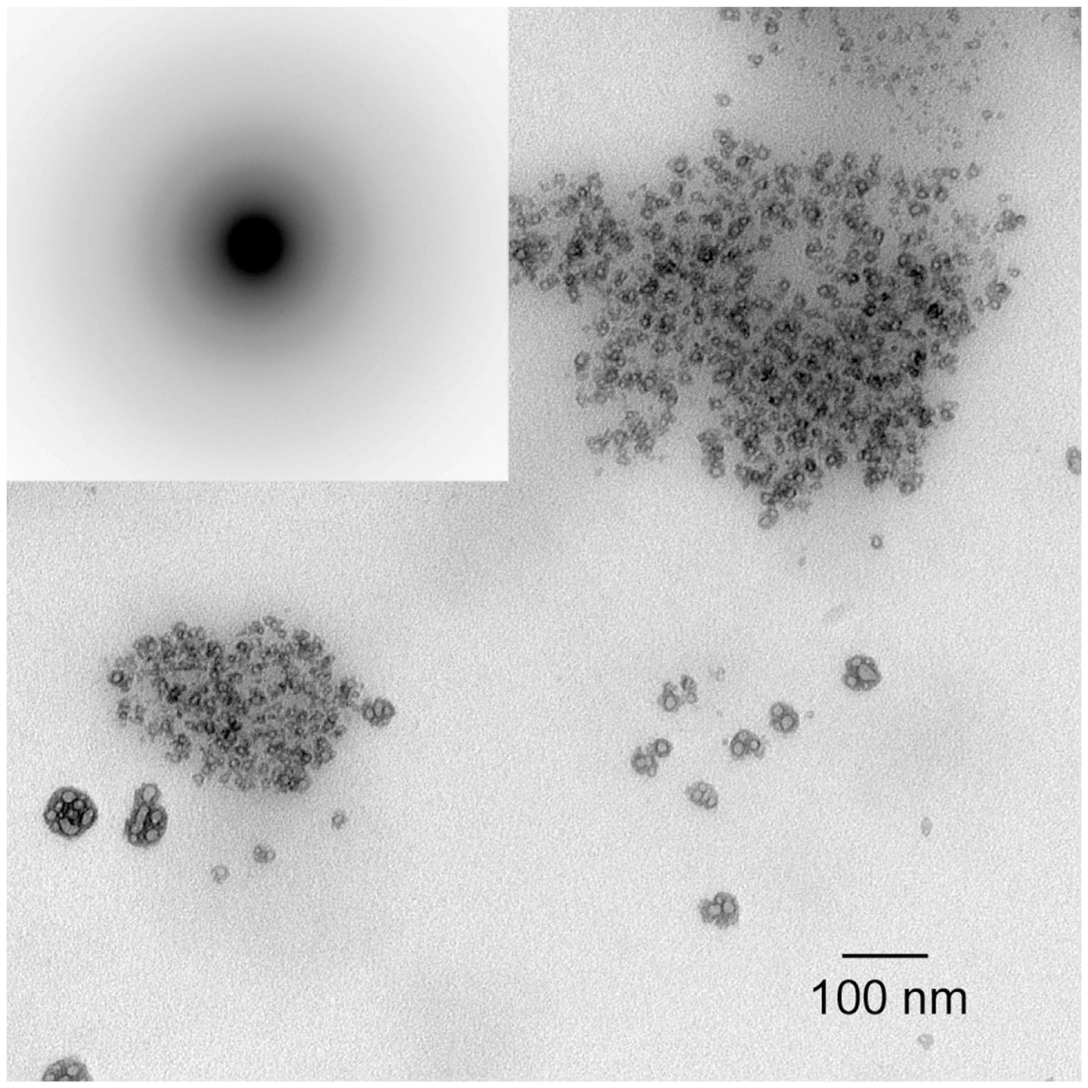
**TEM micrograph of calcium phosphate mineral products formed in the presence of native full-length amelogenin, P173, at 2 mg/mL**. Only ACP was observed, even after 1 d, as confirmed by selected area electron diffraction (inset). This image was reproduced from Wiedemann-Bidlack et al. (2011).

As illustrated in Figure 3, under conditions of relatively high supersaturation brought about by the rapid mixing of calcium and phosphate solutions, mineralization takes place spontaneously. This process involves the *homogeneous* nucleation and formation of precursor ACP nanoparticles, which subsequently transform to crystalline HA. In the absence of a buffer, this process causes a sharp decrease in pH due to the precipitation of a *basic* solid phase (Figure 4). In the presence of the full-length native amelogenin, P173, the solution pH does not drop and remains relatively constant for long periods of time, as illustrated in Figure 4, by preventing the bulk formation of HA crystals, as shown in Figure 5 (Wiedemann-Bidlack et al., 2011). As can be seen in this TEM micrograph, P173 has the capacity to stabilize initially formed nanoparticles of ACP and prevent transformation to crystalline HA, as seen in the control (Figure 3). Of note, truncated native amelogenin, P148 that lacks the hydrophilic C-terminus was similarly found to be a potent stabilizer of ACP (Kwak et al., 2009). Hence, P173 and P148 are inhibitors of spontaneous HA crystal formation. In doing so, the supersaturation status of the experimental solution is also maintained, along with its inherent *stored* capacity to promote mineralization. Notably, native amelogenins like P173 and P148 contain a single phosphorylated site (serine-16) within the N-terminus (Margolis et al., 2006), as further discussed below.

The stabilization of biological fluids through the inhibition of HA crystal formation is well known and appears to fulfill biologically important functions. A prime example is human saliva that has been shown (Hay et al., 1982) to be supersaturated with respect to all known calcium phosphate phases. Evidence suggests that this condition of supersaturation is maintained by a family of salivary phosphoproteins, which have been shown to be potent inhibitors of spontaneous calcium phosphate formation and seeded HA crystal growth *in vitro* (e.g., Hay et al., 1979). Under these conditions that persist in human saliva, calcium phosphate formation on hard and soft tissue surfaces in the oral cavity is prevented, as is the dissolution of tooth enamel in saliva. If saliva did not contain calcium and phosphate ions it would be *undersaturated* with respect to tooth enamel (i.e., with DS_EN_ < 1) and potentially promote the dissolution of enamel surfaces, albeit at a very slow rate. In fact, the observed spontaneous reversal of untreated early carious lesions (Koulourides et al., 1965; Dirks, 1966), where incipient lesions (i.e., white spots) take up mineral ions to become sound surfaces, can be attributed to the condition of saliva supersaturation. Hence, the prevention of unwanted mineral deposits in the oral cavity and the capacity of saliva to promote the repair (remineralization) of beginning enamel lesions is due to resident inhibitors of calcium phosphate formation that maintain saliva in its supersaturated state. Similarly, inhibitors of calcium oxalate formation have been identified in urine and are believed to play an important role in preventing the formation of kidney stones (e.g., Nene et al., 2013). Finally, it has been shown that spontaneous calcification of arteries and cartilage takes place in mice that lack the mineral binding matrix GLA protein, a known mineralization inhibitor. These latter observations, lead the authors of that study to conclude that the calcification of the extracellular matrix is a ubiquitous process that must be actively inhibited in soft tissues that do not normally mineralize (Luo et al., 1997).

### Regulation of biomineral formation

Mann (2001) summation that *“The chemical control of crystallization pathways that involve a sequence of kinetic inhibition and phase transformation can result in a high degree of selectivity in crystal structure and composition,”* is perhaps best illustrated in biology where numerous inhibitors of HA formation have been identified, including magnesium ion, carbonate, proteoglycans, polyphosphate and other phosphorylated molecules. In particular, inorganic pyrophosphate (PP_i_) has been long recognized as a potent inhibitor of mineralization and as a potential regulator of biological mineralization. At micro-molar levels, PP_i_ inhibits the growth (Francis, 1969; Meyer and Nancollas, 1973; Meyer, 1984; Moreno et al., 1987) and dissolution (Fleisch et al., 1966; McGaughey and Stowell, 1977; McGaughey, 1983) of HA, as well as HA crystal formation from ACP (Termine et al., 1970; Robertson, 1973; Blumenthal et al., 1975). A study in our laboratory (Moreno et al., 1987) indicated that the inhibition of HA crystal growth results from PP_i_ adsorption to discrete growth sites on HA surfaces, in the same fashion that has been proposed to explain the inhibition of crystal growth by macromolecules (Moreno et al., 1979). Stabilization of ACP may similarly involve PP_i_ binding to ACP nanoparticles (Ofir et al., 2004).

Based on additional *in vitro* findings (Meyer, 1984) that suggest calcification cannot occur *in vivo* in the presence of physiological levels of PP_i_, it was proposed (Meyer and Reddi, 1985) that pyrophosphatases regulate mineral deposition in bone and cartilage by hydrolyzing PP_i_ (to two molecules of orthophosphate) to eliminate its inhibitory activity. This overall concept has been supported by the elucidation (Ho et al., 2000) of the role of *progressive ankylosis* (*ank*) gene that encodes a transmembrane protein that appears to regulate calcification (and arthritis) by controlling both intracellular and extracellular levels of PP_i_. Additional evidence has also been obtained showing that tissue non-specific alkaline phosphatase (TNAP) and plasma cell membrane glycoprotein-1 (PC-1) are key enzyme regulators of the extracellular PP_i_ concentrations required for controlled bone mineralization (Hessle et al., 2002). PC-1 is the major producer of the PP_i_ inhibitor, while TNAP can regulate the promotion of the onset of bone mineralization by hydrolyzing PP_i_. Consistent with this idea, it has been demonstrated *in vitro* that upon the removal of PP_i_ from solution, either through adsorption or by HA surface-induced hydrolysis (Meyer, 1984; Litman and Margolis, unpublished findings), or by enzymatic degradation of PP_i_, PP_i_-stabilized calcium phosphate solutions rapidly form calcium phosphates.

With respect to amelogenesis, alkaline phosphatase (AP) has been found in secretory enamel, leading investigators to suggest that “dephosphorylation” of amelogenin (e.g., by AP) may play a role in regulating enamel formation (Brookes et al., 1998). Although this suggestion has not been fully substantiated *in vivo*, a study using cultured embryonic tooth germs showed that non-specific inhibition of phosphorylation by casein kinase impaired enamel formation (Torres-Quintana et al., 2000), lending support to the idea that phosphorylated enamel matrix proteins may play a role as mineralization inhibitors. More recently, it has been reported that TNAP is expressed by normal ameloblasts in a stage-specific manner, that is, at the point of transition from secretory to maturation stage (Yadav et al., 2012). In addition, AP was found to be highly expressed by the adjacent stratum intermedium of the developing enamel organ. Importantly, this latter study also showed that the TNAP knockout mice not only exhibited a phenotype similar to infantile hypophosphatasia, but also marked enamel defects, including reduced mineralization and disrupted rod and interrod structures. Hence, the reversal of mineralization inhibition, by PP_i_ hydrolysis or dephosphorylation, may represent a critical regulatory step in the formation of both developing bones and teeth.

Upon mineral growth, macromolecules found in bones and teeth can also effectively influence the *morphology* of calcium phosphate minerals through adsorption to specific crystal planes (e.g., Furedi-Milhofer et al., 1994; Flade et al., 2001). Of particular interest are observations that amelogenin (Iijima et al., 2001, 2002; Beniash et al., 2005), as well as other relevant biomineralization molecules like phosphophoryn (Burke et al., 2000) and glucose (Walsh et al., 1993), preferentially adsorb on (100), (010) faces of HA or OCP, suppressing their growth in a- and b-axis directions. Consequently, under these conditions, such crystals grow preferably along the long c-axis forming ribbon or needle-like structures resembling enamel crystals. As described in the next section, current evidence suggests that native amelogenins have the capacity to stabilize ACP, prevent unwanted crystallization, and ultimately guide the formation and organization of bundles of aligned enamel crystals during the secretory stage of enamel formation. The authors hypothesize, however, that the transformation of ACP to HA and the subsequent growth of HA crystals in thickness and width during maturation are the result of active regulation processes.

### Mechanism of dental enamel formation

**Figure 6:**
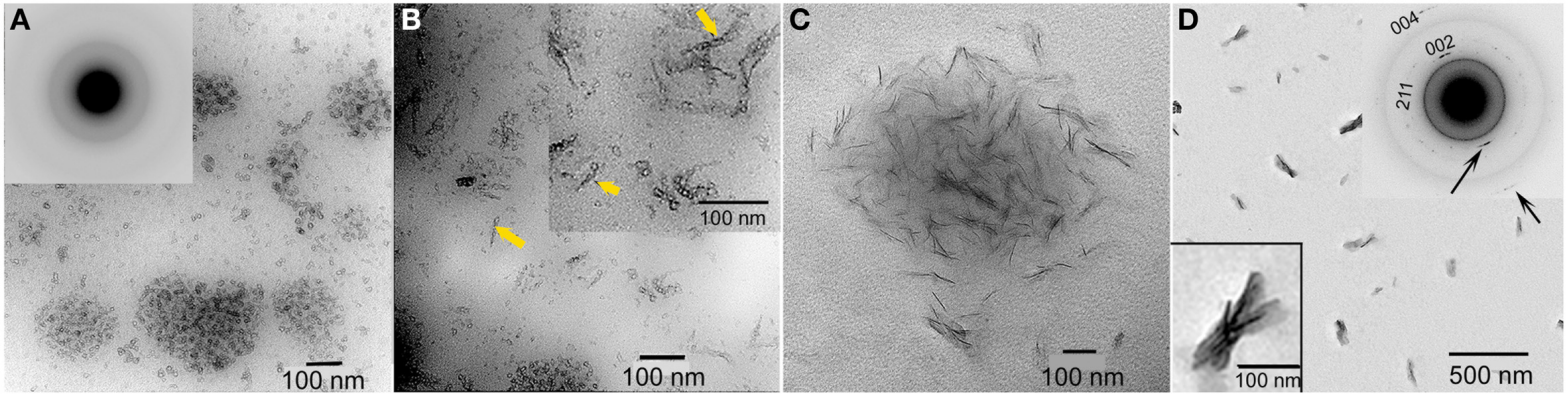
**TEM micrographs of calcium phosphate mineral products formed in the presence of 2 mg/ml recombinant full-length amelogenin, rP172, examined at selected times: 15 min (A), 45 min (B), 1–4 h (C), and 1 d (D)**. ACP particles seen at 15 min (**A**, inset) were found to align to form need-like particles (arrows) at 45 min **(B)**. After 1 d, based on selected area electron diffraction analyzes (**D**, inset), aligned bundles of needle-like apatitic crystals were formed. This image was reproduced from Kwak et al. (2009).

Studies from our laboratory suggest that amelogenin mediates mineralization through a cooperative mechanism where mineralization and protein self-assembly occur simultaneously (Beniash et al., 2005). This conclusion is based on *in vitro* findings that show that the non-phosphorylated full-length recombinant mouse amelogenin, rM179, can guide the formation of well-aligned bundles of HA crystals *in vitro*, under experimental conditions designed to simultaneously induce amelogenin assembly and mineralization. Organized mineral formation was not observed when protein self-assembly was carried out prior to mineralization or when a truncated recombinant mouse amelogenin (rM166, lacking the hydrophilic C-terminus) was used. Similar results were subsequently obtained under cooperative assembly/mineralization conditions using recombinant full-length porcine amelogenin, rP172, which was also found to guide the formation of bundles of aligned HA crystals (Kwak et al., 2009). These latter experiments were monitored as a function of time to provide insight into the mechanism of amelogenin-mediated mineralization. These studies revealed that rP172 transiently stabilizes ACP nanoparticles (Figure 6), which were found to be significantly smaller than those seen in the control (Figure 3). As the reaction continued in the presence of rP172, an alignment of ACP nanoparticles was observed at ~45 min. Arrays of needle-like crystals were then observed between 1–4 h. At 24 h, as shown, multiple bundles of well-aligned needle-like apatite crystals were seen that are similar to those found in the presence of rM179 (Beniash et al., 2005) and distinctly different in comparison to the randomly arranged plate-like crystals found in the control (at 24 h, Figure 3). Truncated recombinant (non-phosphorylated) porcine amelogenin rP147 had relatively little effect on the mineralization process (Kwak et al., 2009), again indicating that the full-length amelogenin has a unique capacity to guide ordered mineralization. This unique capacity is attributed to the fact that full-length recombinant mouse (rM179) and porcine (rP172) amelogenin, as well as full-length native amelogenin (P173), undergo self-assembly to form hierarchical nanosphere (Fang et al., 2011, 2013) and chain-like (Wiedemann-Bidlack et al., 2007, 2011) structures, as a function of solution pH. Truncated amelogenins lacking the hydrophilic C-terminus do not form such organized structures. Notably, the single phosphate group in native amelogenins on serine-16 was found to have only a subtle influence on P173 assembly and structure, as determined by both conventional (Wiedemann-Bidlack et al., 2011) and cryo-TEM (Fang et al., 2013). However, small-angle X-ray scattering studies using phosphorylated and non-phosphorylated leucine-rich amelogenin peptides (LRAP, an alternative splice product that is comprised of the first 33 amino acids and the last 23 C-terminal amino acids of full-length (porcine) amelogenin) showed that phosphorylation of serine-16 had a significant effect on the folding of the peptide in solution (Le Norcy et al., 2011a). LRAP(+P) exhibited an unfolded structure, while LRAP(-P) exhibited a globular structure in solution. LRAP(+P), like P173, was also found to effectively stabilize ACP and inhibit HA formation *in vitro* (Le Norcy et al., 2011b). These influences on protein structure and folding may be of considerable functional importance, given the marked effect phosphorylation has on the capacity of native amelogenins to stabilize ACP and inhibit HA formation for extended periods of time, as further discussed below.

**Figure 7:**
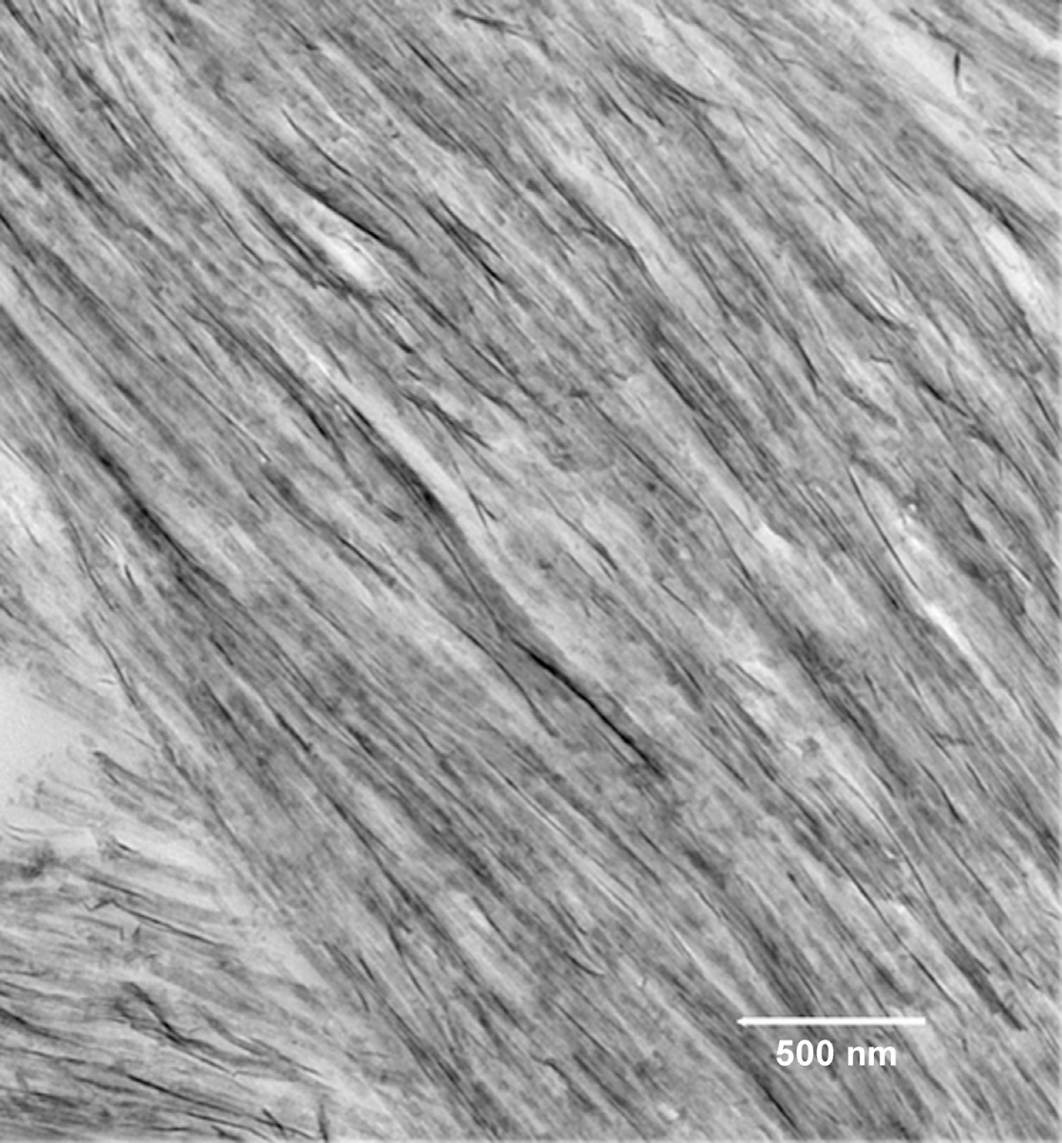
**Transmission electron microscopy micrograph of secretory stage mouse enamel showing parallel arrays of long thin ribbons of mineral**.

In recent years, two prominent theories of crystal growth—aptly named the classical and non-classical crystal growth theories, have received considerable attention, in part, through detailed investigations into the growth mechanisms of biominerals (Niederberger and Colfen, 2006). The classical mechanism for crystal growth is mineral ion mediated where mineral growth progresses through the successive accumulation of mineral ions to form a single crystal. The non-classical mechanism for crystal growth is mediated by initially formed nano-particles that subsequently assemble in an organized fashion to form what appear to be single crystals (so-called mesocrystals). Although it is often difficult to distinguish between these two mechanisms, early studies (Robinson et al., 1981; Robinson, 2007) have suggested that, during amelogenesis, enamel crystals form through the assembly of small amorphous mineral particles, although the amorphous nature of these particles was not verified experimentally. Since this initial proposal, however, data have been obtained that support the presence of ACP in the developing enamel matrix (Diekwisch, 1998; Bodier-Houlle et al., 2000). In a more recent study (Beniash et al., 2009), multiple independent techniques were used to carefully characterize the phase of the long thin parallel arrays of mineral deposits (e.g., as seen in Figure 7) that form during the secretory stage of amelogenesis that begin near the DEJ and extend to the enamel surface. Newly deposited mouse enamel, that is, in the outer enamel layers closer to the enamel surface and the ameloblast layer was found to be ACP, while older enamel, found in inner layers of enamel closer to DEJ contained apatitic crystals. Of particular importance, the newly deposited amorphous mineral and the older crystalline deposits had the same ribbon-like shape and organization in parallel arrays. These findings suggest that enamel mineral morphology and organization are established by the enamel matrix prior to the transformation of ACP to HA-like enamel crystals. These findings are also consistent with a proposed hypothesis that initial enamel crystals form from the assembly and fusion of amorphous calcium phosphate particles (e.g., Robinson, 2007). Transient amorphous mineral phases (i.e., ACP and amorphous calcium carbonate) have also been observed to be associated with a number of other biomineralization systems (Lowenstam and Weiner, 1985, 1989; Beniash et al., 1997; Wu et al., 1997; Levi-Kalisman et al., 2000; Aizenberg et al., 2002), including bone (Kim et al., 2005; Crane et al., 2006; Mahamid et al., 2008), leading to the proposal that the transformation of amorphous phases to crystalline materials is an important aspect of biological mineralization (Beniash et al., 1997; Weiner et al., 2005). Other molecules believed to be associated with the regulation of biomineralization, like highly phosphorylated osteopontin from milk (Gericke et al., 2005) and the N-terminal domain of dentin matrix protein 1 (Gajjeraman et al., 2007) have been shown to stabilize ACP.

### Potential role of mineralization inhibitors in dental enamel formation

**Figure 8:**
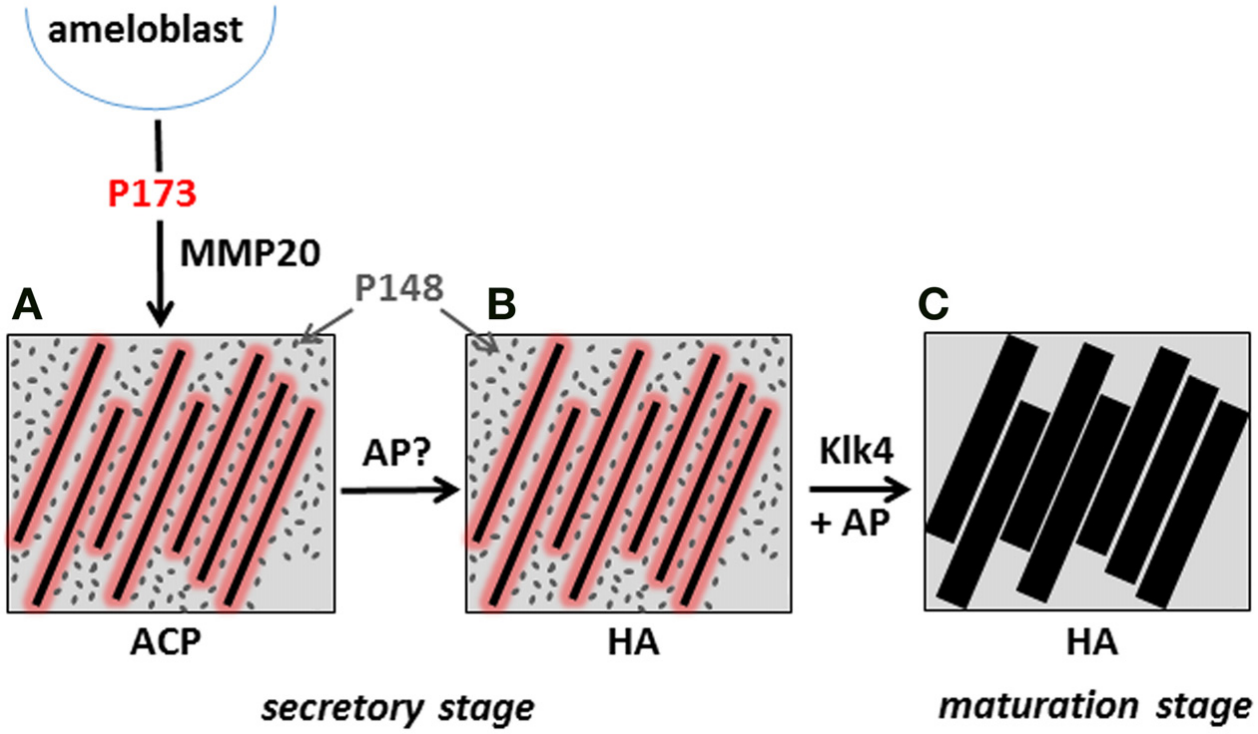
**Proposed model illustrating the potential role of P173 and P148 in dental enamel formation**. The onset of the initial formation of linear arrays of ACP **(A)** during the secretory stage is brought about by the marked reduction of P173 by MMP20, reversing its inhibitory effect. Limited quantities of P173 (red) stabilize linear ACP arrays through direct interaction, as illustrated. The predominant P148 (gray) cleavage product plays a key role in preventing unwanted mineralization, while maintaining conditions of supersaturation. The linear arrays of ACP subsequently transform to apatitic crystals (Beniash et al., 2009) during the secretory stage **(B)**. During maturation, inhibition of bulk mineralization provided by P148 is eliminated by Klk4 proteolysis, along with small amounts of adsorbed P173 growth inhibitor, to initiate the lateral growth of initial enamel ribbons **(C)**. As noted in the text, AP may also play an essential role in regulating mineral formation in the secretory stage and mineral growth during the maturation stage by catalyzing amelogenin dephosphorylation.

Based on current evidence, inhibition of mineralization by native amelogenins and its reversal *in vivo* may play critical roles in enamel formation. This conclusion is supported by data showing (1) an inverse relationship between protein and mineral content (Figure 1), and the notably high protein and low mineral content (10–20% by volume) of secretory stage enamel; (2) the capacity of native amelogenins to stabilize ACP and inhibit mineralization *in vitro*; (3) the apparent requirement for both proteases [i.e., matrix metalloproteinase 20 (MMP20) during the secretory stage Caterina et al., 2002 and kallikrein 4 (Klk4) during transition/maturation Hu et al., 2000; Simmer et al., 2009] and phosphatases (i.e., AP, as discussed above) for proper enamel formation; and (4) the rapid onset of mineral growth coinciding with loss of protein (Figure 1). These collective data suggest that during the secretory stage of enamel formation, enamel matrix proteins selectively guide ACP nanoparticle organization and transformation to thin ribbons of co-aligned HA crystals, while at the *same time* preventing unwanted bulk mineralization. Support for this scenario was recently provided by *in vitro* findings showing that under the proper kinetic conditions, controlled by variations in protein concentration, significantly lower concentrations of P173 can guide the transformation of initially stabilized ACP nanoparticles to ordered bundles of HA crystals that have similar morphology to initially formed enamel crystals (Kwak et al., 2014). In contrast to that seen with P173, however, P148 lacked the capacity to regulate ordered mineralization at reduced protein concentrations, although mineralization did take place. At higher concentrations, again, both P173 and P148 are potent stabilizers of ACP. Hence, upon its secretion, the parent phosphorylated full-length amelogenin P173 can initially prevent premature mineralization of HA crystals in forming enamel. However, full-length amelogenin undergoes proteolytic processing soon after secretion, resulting in a significant *decrease* in its concentration. In developing porcine enamel, the P173 content of secretory enamel was found to be only 7.4% of total amelogenin, a reduction in P173 by more than 90% (Wen et al., 1999). P173 was also found to be exclusively associated with newly formed enamel mineral (Uchida et al., 1991). In contrast to P173, the P148 content of the enamel matrix *increases* and becomes the predominant amelogenin cleavage product at 49.5% of total amelogenin (Wen et al., 1999). Based on these combined *in vitro* and *in vivo* findings, the authors propose that the onset of enamel crystal formation is regulated by a marked reduction (>10-fold) in P173 concentration to attain a critical lower level that has the potential to organize forming mineral deposits into oriented bundles of elongated HA crystals, as illustrated in a proposed model presented in Figure 8. At the same time, substantially higher concentrations of P148 (~ 6–7 times higher than P173) that accumulate in the developing (porcine) enamel matrix serve an important role by preventing uncontrolled mineralization throughout the secretory stage of amelogenesis where the mineral volume is only 10–20% (Robinson et al., 1988; Fukae, 2002).

The noted proteolytic processing of amelogenin during the secretory stage of amelogenesis has been primarily attributed to MMP20 that is exclusively expressed during this initial stage of enamel formation. MMP20 has also been shown to be essential for proper enamel formation (Caterina et al., 2002). In its absence, the enamel layer is about one-half the normal thickness and the decussating prism pattern of normal enamel is substantially disrupted. These latter findings verify that proteolytic processing of full-length amelogenin is required for proper enamel formation, consistent with our proposed model, that is based on the capacity of reduced levels of P173 to guide ordered HA crystal formation, while accumulating P148 levels maintain mineralization driving forces by preventing unwanted bulk mineralization. During maturation, Klk4 is predominantly expressed and is believed to be responsible for removing almost all of the remaining enamel matrix to allow the initial enamel crystals to grow in width and thickness (Hu et al., 2000). Hence, as illustrated in the presented model, we propose that the onset of mineralization during the secretory stage is brought about by the marked reduction of P173 content by MMP20, reversing its inhibitory effect. During maturation, inhibition of bulk mineralization provided by P148 is eliminated by Klk4 proteolysis, along with small amounts of adsorbed P173 growth inhibitor, to initiate the lateral growth of the secretory stage enamel ribbons. As noted above, AP may also play an essential role in regulating mineral formation in the secretory stage and mineral growth during the maturation stage by catalyzing amelogenin dephosphorylation and removing the noted mineralization inhibitory activity found to be associated with native amelogenins. The timing of the expression of key regulators of biomineralization during enamel formation is, therefore, of critical importance (e.g., Smith, 1998; Simmer et al., 2009). Additional studies addressing the potential roles of amelogenin proteolysis and de-phosphorylation in regulating initial enamel mineral formation and maturation are currently underway in our laboratory.

## Conclusions

Mineralization inhibitors can play critical roles in regulating mineralized tissue formation, stability, and regeneration *in vivo*. With respect to dental enamel formation, mineralization events triggered by the proteolysis and/or de-phosphorylation of full-length native amelogenin during the secretory stage of amelogenesis may play an essential role in regulating enamel mineral formation and its hierarchical structure, while predominant amelogenin degradation products, like P148, serve to prevent uncontrolled mineral formation. During maturation, residual inhibition is removed via additional matrix processing, allowing initial enamel crystals to expand to form the highly dense enamel mineral layer.

### Conflict of interest statement

The authors declare that the research was conducted in the absence of any commercial or financial relationships that could be construed as a potential conflict of interest.
